# What Do NAFLD, Liver Fibrosis, and Inflammatory Bowel Disease Have in Common? Review of the Current Literature

**DOI:** 10.3390/metabo13030378

**Published:** 2023-03-03

**Authors:** Sara Jarmakiewicz-Czaja, Jolanta Gruszecka, Rafał Filip

**Affiliations:** 1 Institute of Health Sciences, Medical College of Rzeszow University, 35-959 Rzeszow, Poland; 2 Department of Clinical Microbiology, Clinical Hospital No. 2, 35-959 Rzeszow, Poland; 3 Institute of Medicine, Medical College of Rzeszow University, 35-959 Rzeszow, Poland; 4 Department of Gastroenterology with IBD Unit, Clinical Hospital No. 2, 35-959 Rzeszow, Poland

**Keywords:** biomarkers, Crohn’s disease, inflammatory bowel diseases, liver fibrosis, nonalcoholic fatty liver disease, ulcerative colitis

## Abstract

Liver disease is one of the most common extraintestinal manifestations of inflammatory bowel disease (IBD). Often the course of liver disease is associated with an exacerbation of the underlying disease (Crohn’s Disease/Ulcerative Colitis). Nonalcoholic steatohepatitis encompasses a wide spectrum of liver damage. The most common form is nonalcoholic fatty liver disease (NAFLD) (75–80%), and the less common but more dangerous form is nonalcoholic steatohepatitis (NASH). NAFLD is now the most common cause of chronic liver disease in developed countries and the leading indication for liver transplantation in the United States. Genetic, demographic, clinical, and environmental factors can play a role in the pathogenesis of NAFLD. The increasing prevalence of NAFLD is associated with a widespread obesity epidemic, metabolic complications, including hypertension, type 2 diabetes, and dyslipidaemia. Some of the most common manifestations of IBD are liver, biliary tract, and gallbladder diseases. The liver fibrosis process has a complex pathophysiology and is often dependent on exogenous factors such as the treatment used and endogenous factors such as the gut microbiome. However, the factors that link IBD and liver fibrosis are not yet clear. The main purpose of the review is to try to find links between IBD and selected liver diseases and to identify knowledge gaps that will inform further research.

## 1. Introduction

Chronic liver disease (CLD) is one of the leading causes of mortality, morbidity, and the use of healthcare resources around the world [1]. From 1980 to 2010, CLD-related mortality increased by 46% worldwide [2]. This increase was observed mainly in low- and middle-income countries in Asia and Africa [3]. The factors that contribute to the increase in mortality vary in different parts of the world. In a recent study conducted in the United States (USA), an increase in mortality from liver disease was associated with an increased prevalence of nonalcoholic fatty liver disease (NAFLD) [4]. NAFLD is a liver disease associated with obesity, insulin resistance, type 2 diabetes mellitus (DM2), hypertension, hyperlipidemia, and metabolic syndrome. A subtype of NAFLD, which is histologically classified as nonalcoholic steatohepatitis (NASH), has a potentially progressive course leading to liver fibrosis, cirrhosis, hepatocellular carcinoma (HCC) and liver transplantation [5]. NASH is characterized by lobular inflammatory infiltrates, ballooning hepatocytes (hepatocytes, the most abundant liver cells, swell up to twice their normal size, which heralds their death) and cell death, fibrosis, and ultimately cirrhosis [6,7]. All of these complications of NASH can pose a significant burden on health, economic, and experience in patients, their families, and society [5]. Liver diseases are one of the most common manifestations of extraintestinal inflammatory bowel diseases. IBDs are chronic diseases that occur with periods of active phase and remission. Often, the course of liver disease is related to the activity of the underlying entity (Crohn’s Disease/Ulcerative Colitis). Organ inflammation can be caused by some treatments with IBD [8]. It is important to know the links between liver disease and IBD due to the fact that a persistent inflammatory process that takes place in the liver can cause fibrosis, cirrhosis, or liver cancer [9].

## 2. Nonalcoholic Fatty Liver Disease (NAFLD) and Liver Fibrosis (LF)

NAFLD is the most common chronic liver disease. Its increasing prevalence is due to high-calorie diets and sedentary lifestyles. Central obesity, insulin resistance, and DM2 are strong independent risk factors [6]. The prevalence of NAFLD is proportional to increasing Body Mass Index (BMI) [5]. It is about 25% in the general population, but increases to more than 90% in highly obese individuals undergoing weight loss procedures and surgeries [5]. As age increases, the prevalence of NAFLD and NAFLD-related fibrosis increases [10]. In addition to the higher incidence of NAFLD, a higher degree of fibrosis has been observed in the elderly. This may be due to the increased prevalence of metabolic diseases in this group of individuals [11]. Additional studies have confirmed the association of age with a higher risk of severe liver fibrosis, HCC, and DM2 [12]. There is also evidence that up to 27% of NAFLD cases may be associated with familial predisposition [13]. The main factors most strongly associated with the progression of NAFLD include older age (although this may be more related to the duration of exposure than to ordinary age), the presence of visceral obesity, the prevalence of DM2 or insulin resistance [14]. A meta-analysis of 33 studies found an association between NAFLD and a 2-fold increase in the risk of chronic kidney disease (CKD) [15].

Liver fibrosis is a process caused by chronic exposure to damaging agents: drugs, viruses, alcohol. Alcohol-related liver disease can be considered in men who consume more than 30 g of alcohol/day and women who consume more than 20 g of alcohol/day [16]. Fibrosis is related to the repair functions of liver cells, whose balance is disrupted. In the initial phase, it is a reversible process, but with significant damage a chronic disease such as cirrhosis develops [17,18,19]. When the liver is exposed to harmful agents or chronic inflammation, degenerative processes result in the destruction of damaged cells and their replacement by collagen with the formation of scar tissue. Fibrosis is a mechanism that maintains the integrity and strength of the organ despite the death of its cells. Fibrosis is not a constant and continuous process; it can have periods of exacerbation and extinction. The final phase of fibrosis leads to cirrhosis and irreversible liver damage. In an advanced state of cirrhosis, the patient’s only chance is a liver transplant. If cirrhosis persists for a long time, primary liver cancer can develop [17,18].

The mechanism of liver fibrosis of NAFLD is complex. In the first stage, due to increased lipolysis of visceral adipose tissue (AT), an excessively high supply of fats and calories in the daily diet, and liver de novo lipogenesis (DNL), fat accumulation occurs in hepatocytes. Disturbances in lipid metabolism can lead to the formation of lipotoxic lipids, which can result in cellular stress because the cell’s ability to oxidise fatty acids is too low relative to the increased amount free fatty acids (FFA), thereby increasing reactive oxygen species (ROS) production. The increased amount of FFA also negatively affects other factors, e.g., up-regulates mitochondrial respiration. The development of NASH from NAFLD occurs due to, among other things, the activation of molecules from AT lipolysis secretion and intestinal barrier dysfunction. Thus, activating macrophages and promoting activation of hepatic stellate cells (HSCs) leads to increased extracellular synthesis [20,21,22]. HSCs can be activated by increased ROS production [23]. Furthermore, Toll-like receptors (TLRs) are factors that mediate HSC activation. In turn, activated HSCs secrete transforming growth factor β1 (TGFβ1) and tumor necrosis factor α (TNFα), which predispose to increased inflammation and collagen deposition, leading to the progression of organ fibrosis [24,25].

Among the causes of liver fibrosis are alcohol abuse, drug intoxication (chronic use of painkillers and anti-inflammatory drugs, as well as highly toxic drugs such as methotrexate), biliary tract diseases (primary sclerosing cholangitis-PSC), viral infections (e.g., hepatitis C), autoimmune diseases, metabolic diseases (hemochromatosis, Wilson’s disease), hepatic steatosis, and parasites [18]. The following factors influence the rate of liver fibrosis: alcohol consumption (regardless of type, more than 50 g of ethanol per day), treatment of autoimmune diseases, liver steatosis, age (>40 years), diabetes, obesity. The biggest problem with liver fibrosis is that in mild to moderate states it produces virtually no symptoms, so it does not require the sufferer to seek help. Only in the most advanced states do liver failure and associated symptoms occur, including nausea, vomiting, lack of appetite, increased abdominal girth, swelling of the extremities, weakness, and decreased intellectual performance [17,19].

As part of the basic assessment of liver function and efficiency, blood tests (alanine aminotransferase-ALT, aspartate aminotransferase-AST, alkaline phosphatase-ALP, gamma-glutamyl transferase-GGT, bilirubin, urea, ammonia) can be used, informing about possible liver cell damage or bile stasis, and metabolic efficiency (whether the liver is able to neutralise toxins in our body). Abdominal ultrasound is also a valuable test, determining the “density” of the liver parenchyma and the possible presence of focal lesions (tumours, abscesses) within the liver. However, it has its drawbacks–it is an invasive procedure, has its complications, and numerous contraindications (such as coagulation disorders). Another test with efficacy comparable to biopsy, but without skin puncture, is dynamic elastography [17,18].

In treating the liver, the most important thing is to avoid hepatocyte damaging agents, giving up alcohol, psychoactive drugs, and limiting the use of painkillers and anti-inflammatory and hepatotoxic drugs. This is the only currently known method to efficaciously suppress liver fibrosis and prevent cirrhosis. The basis of liver health is proper diet recommendations. Physical activity and weight reduction have a positive effect on liver function. The diet recommendations for liver disease include: exclusion of alcohol, consumption of 5–6 smaller meals per day, consumption in peace, without stress, thorough chewing, consumption of cooked or baked in foil, limitation of fat intake to 50 g per day, exclusion of hard to digest, bloating and waspish spices, consumption of easily digestible foods–light bread, white rice, lean dairy products, vegetable oils, boiled potatoes, boiled vegetable etables (cabbage vegetables, onions, chives, radishes, beans, peppers are excluded), herbs (dill, parsley, cumin), still water [18,19].

## 3. Inflammatory Bowel Diseases (IBD)

**Figure 1:**
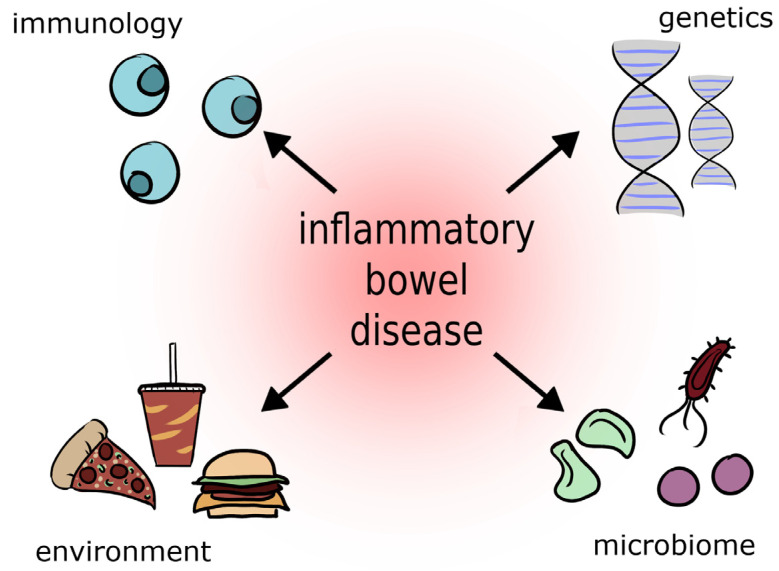
Etiology of Inflammatory Bowel Disease [27,31].

IBD cause chronic inflammation and can affect various sections of the gastrointestinal tract. A particular form of inflammatory bowel disease is inflammatory bowel disease (IBD) [26]. It is estimated that about 2.5–3 million people in Europe struggle with this disease. The prevalence of inflammatory bowel disease in the United States is 70 to 150 cases per 100,000 people and is increasing [27]. In Poland, approximately 50,000 people suffer from inflammatory bowel disease, one in four of whom are minors [26,28]. Inflammatory bowel diseases include CD, UC, indeterminate colitis, and microscopic colitis [26,29,30]. Among the causes that influence the onset of IBD are genetic, immunological, and environmental factors (dietary changes, stress, overuse of antibiotics, especially in the first years of life, smoking, alcohol, progressive environmental degradation, high content of artificial preservatives in food) [27,31] (Figure 1).

An example of immune factors predisposing to IBD is an imbalance of Th17/Treg cells. This balance is influenced by the gut microbiome, T Cell Receptor (TCR) signaling or cytokines, among other factors [32]. Furthermore, changes in the number of T cells can also lead to the development of LF, for example, by indirectly inducing liver damage via Th17 and Tc (cytotoxic T) cells [33]. Many authors also point out intestinal homeostasis disturbances in patients with IBD (Table 1). These conditions affect people of different ages. According to statistics, most patients are adults between the ages of 20 and 40, but a third of patients are adolescents and children. IBD is increasingly affecting older people. Infants are the least likely to suffer from inflammatory bowel disease [26].

### Symptoms and Diagnostics of Inflammatory Bowel Disease

People with inflammatory digestive disease often experience abdominal pain, nausea, and appetite disorders. Vomiting, diarrhoea, and a subfebrile state are common. These symptoms cause impaired absorption, leading to nutrient deficiencies and hypovitaminosis [45]. Among patients diagnosed with chronic IBD, the incidence rate of depression is 15–30%, with up to 80% of patients experiencing anxiety during disease exacerbation compared to fully healthy individuals. This is caused by the deterioration of quality of life by interfering with normal physical and mental functioning. Patients rarely need psychological support, which is an important part of a holistic approach to their care [46]. IBD sufferers often exclude various foods from their diet, identified with their ailments. Because of this, they may experience hypoproteinemia and low levels of vitamins. In serum, A, D, K, C and B have low concentrations of minerals, iron, zinc, magnesium, resulting in reduced immunity, problems with wound healing, and more frequent infections occur. Inflammatory bowel diseases increase the risk of osteoporosis and osteopenia as a consequence of vitamin D and calcium deficiency, which affect between 3% and 30% of patients [45]. Inflammatory bowel disease leads to serious and life-threatening complications ranging from intestinal perforation, joint, skin, eye, and liver disease to colon cancer [45].

UC is one of the diseases of the IBD. It involves inflammation of the mucosa of the colon, leading to widespread and shallow ulcerations. Symptoms: diarrhoea, mucus, and blood fragments may appear, abdominal cramps and pain, sudden feeling of pushing on the stool, lack of appetite, significant weight loss. Occasionally, UC often produces extraintestinal symptoms, such as uveitis and scleritis, joint pain, erythema nodosum, alopecia, liver disease, thrombosis, and anemia [26,27,47].

Another disease of inflammatory bowel disease is CD. It is chronic and progresses slowly. Inflammation occupies the intestinal mucosa partially and sometimes even entirely, leading to fistulas, abscesses, and ulcers. The most common symptoms are abdominal pain and cramps of varying severity, vomiting, diarrhoea, nausea, flatulence, and extraintestinal symptoms.

Both of these conditions prevent normal functioning. Patient reports indicate that they sometimes use the bathroom up to 20 times a day [26,27,47].

The diagnosis of IBD is based on invasive and noninvasive methods. For the proper diagnosis of IBD, an endoscopic examination, gastroscopy, and colonoscopy must be performed, which requires adequate preparation, often causing stress and anxiety in patients. It is contraindicated in cases of exacerbation of the disease [48]. Sometimes a histopathological examination of the specimen is necessary. This is followed by a radiographic examination or magnetic resonance imaging of the intestines.

Determining the biomarkers calprotectin and lactoferrin in the stool is helpful for diagnosing IBD [31]. These proteins are released into the gastrointestinal tract as a response to intestinal inflammation. Calprotectin is secreted by immune cells located in the deeper layers of the intestine [45,49,50,51]. The test of a stool sample for the presence of lactoferrin protein also provides valuable information on intestinal status. Lactoferrin is produced by mucosal epithelial cells and is crucial for normal mucosal defence of the gastrointestinal tract [52,53]. Elevated fecal lactoferrin levels signify an increased mucosal immune response to food or bacterial antigens and may indicate a chronic state of inflammatory gastrointestinal disease. If this is the case, a further diagnosis is recommended [49,52,53,54].

IBD have in common that disease exacerbations alternate with moments of remission, when symptoms resolve completely or partially. Treatment should be tailored to the stage of the disease, the patient’s condition, and the specific symptoms present. Complete cure is not possible, so therapy consists of pharmacological maintenance of the remission state and alleviation of symptoms [49,50,51,55].

## 4. Primary Sclerosing Cholangitis (PSC) and Inflammatory Bowel Disease (IBD)

PSC is a progressive chronic cholestatic disease that can lead to biliary fibrosis, biliary stricture, and eventually liver disease such as cirrhosis [56]. The substrate of PSC can be genetic, where with a positive family history of PSC, the risk of the disease is 10–20 times higher than in the general population [57].

Through the Genome-Wide Association Study (GWAS), 23 susceptibility loci for PSC-IBD have been identified [58]. Bacterial translocation and subsequent portal tract bacteremia are also important risk factors. [59] Additional risk factors can include chronic viral infections and ischemic vascular injury [60]. Eksteen’s work points to the breakdown of the intestinal-hepatic axis microbiome in addition to immunological and genetic factors as a cause of PSC-IBD [61]. Confirmation of the link between the gut microbiome and PSC may be the positive therapeutic Response of PSC with vancomycin [62]. The authors of several studies on the relationship between PSC and IBD conclude that the composition of the gut microbiota of patients with PSC-IBD is different from that of patients with IBD without co-occurring PSC [63,64]. Mehta et al. in their meta-analysis present that PSC is present in approximately half of patients with IBD on average, more often with a diagnosis of UC compared to CD [65,66]. However, in many publications, the prevalence varies, which may be due to geographical differences related to the place of residence of the patients [67]. Some studies suggest that men with UC are more likely to develop PSC than women. PSC in patients with IBD is usually asymptomatic or the disease has a mild course. The most common symptoms reported by patients are pain, fatigue, and wheezing. Treatment depends on the symptoms and complications of the disease (present strictures with symptoms in the course of PSC, endoscopy, advanced PSC leading to cirrhosis, liver transplantation) [60]. In a Danish retrospective study, the authors indicate that patients with PCS-IBD are treated more intensively than patients with IBD alone [68]. Ricciuto et al. in their article indicate that PSC-IBD has a unique disease phenotype [69]. It is typically characterized by benign chronic inflammation with localization on the right side, but with an increased risk of colorectal cancer [67,70,71]. Researchers often treat PSC-IBD as a separate disease entity. Furthermore, some studies have reported that colectomy performed in patients with IBD before PSC diagnosis may be associated with a reduced risk of liver transplantation, but more research is needed on this topic [72]. The pathogenesis of PSC-IBD is not completely clear. Some researchers hypothesize that the cause may be T cells in the gut, which with the expression of CCR9 (CC-chemokine receptor 9) and integrin α4β7 show the ability to migrate to the liver. This may happen, among other things, due to aberrant expression of the adhesion mucosal vascular addressin cell adhesion molecule 1 (MAdCAM-1) [73]. One of the less explored links is the occurrence of PSC-IBD with liver fibrosis. Hirschfield et al. in their article present that 7 loci are associated with IBD, PSC and liver fibrosis [74]. Increasingly, researchers are pointing to a direct link between IBD and liver fibrosis.

## 5. Liver Fibrosis and Inflammatory Bowel Disease

The liver fibrosis process has a complex pathophysiology and often depends on exogenous factors, such as the treatment used, and endogenous factors, such as the gut microbiome. However, the factors that link IBD and liver fibrosis are still unclear [75,76,77].

### 5.1. Studies in Humans

Studies in humans that define the link between liver fibrosis and IBD are still rare. In one study, Ritaccio et al. examined the prevalence of NAFLD and liver fibrosis in patients with IBD. Researchers assessed the severity of liver fibrosis using the NAFLD fibrosis score (NFS). They observed that the risk of liver fibrosis in patients with CD or UC is low. Furthermore, they indicate that probable fibrosis in patients with IBD is independent of pharmacotherapy [78]. In a study by Barbero-Villares et al. advanced liver fibrosis (F ≥ 3) was found in 6.5% of patients with IBD, while F = 2, in 17.4%, but only methotrexate-treated patients were included in the study [79]. A similar study by Laharie et al. was conducted in which the researchers also found that cumulative doses of methotrexate (of more than 1500 mg) rarely cause liver fibrosis in patients with IBD [80]. However, patients with methotrexate-treated IBD have a statistically significantly higher incidence of liver damage, including LF, compared to patients treated with the same drug with diseases other than IBD [81]. In another study, Magri et al. found liver fibrosis (F ≥ 2) in 16% of patients with IBD. In addition, they found no risk factors associated with IBD or LF. Instead, they showed a statistically significant association between MetS (metabolic syndrome) and LF, and identified risk factors as the same as in the general population [82]. Carr et al. reached similar conclusions, showing that liver disease (NAFLD) is associated with MetS, but not with the course of IBD [83]. In a large cross-sectional study that included 831 patients for analysis, in which the authors examined the prevalence of metabolic-associated fatty liver disease (MAFLD) and LF and their risk factors in patients with IBD, the authors observed that MAFLD and advanced liver fibrosis were more common in patients with IBD than in healthy subjects. Furthermore, the researchers identified potential risk factors for advanced liver fibrosis in MAFLD, such as BMI above normal, type 2 diabetes, high blood pressure, and IBD. In addition to the risk factors mentioned above for MAFLD, the study authors also found that a history of complications from IBD and older age were important factors in the development. Furthermore, patients with CD are at increased risk of developing MAFLD and its sequelae, such as LF [84]. Veltkamp et al. also indicate that liver fibrosis is more common in patients with CD compared to patients with UC [85].

Intestinal fibrosis is also common in IBD. Liver and intestinal fibrosis can occur by activating mesenchymal cells. Another common factor is the activation of Toll-like receptors (TLRs) by translocation of bacteria and/or their products [86]. Furthermore, Inagaki et al. point to transforming growth factor β (TGF-β) as a cytokine that plays an important role in the emerging fibrosis process [87]. Components that activate fibroblasts and increase extracellular matrix (ECM) production include interleukins, for example, IL-1, IL-6, IL-21, while IL-10 and IL-12 demonstrate the opposite effect. Therefore, studies are underway to incorporate therapies, such as IL-10, to reduce the rate of progression of fibrosis or inhibit it [88,89].

### 5.2. Animal Studies

In a study in animal models, Baumann et al. tested whether the change in intestinal microflora composition in mice is associated with degeneration of the intestinal barrier and the appearance of inflammation and liver fibrosis. Due to the age of the animals, changes occurring in older individuals due to a change in the intestinal microflora may have been caused by a change in the expression of antimicrobial peptides and inducible nitric oxide synthase (iNos). In the 24 and 30 month old mice studied, normal intestinal barrier function was reduced compared to the younger animals. The authors also observed multiple inflammatory foci in the livers of the animals. Bacterial endotoxins could also be related to Lbp expression. Furthermore, they observed an increase in the expression of TLR 1, 4, 9 (toll-like receptors–the protein family is important in nonspecific immune response) among others, in the liver, which may be related to the onset of organ fibrosis [90]. In another study, Xie et al. investigated changes in the gut microbiota during liver disease. The study was carried out in animal models in which liver disease was induced with a high-fat streptozotocin diet (STZ-HFD). They found liver fibrosis to occur in 12 week old mice. They showed that *Firmicutes* abundance gradually increased with liver damage, while *Bacteroidetes* abundance decreased [91]. *Bacteroidetes* are also found in a reduced number of patients with IBD, while no increase in *Firmicutes* is observed [92]. Patients with liver disease, such as cirrhosis of this organ, have significant intestinal dysbiosis, where *Fusobacteria* and *Proteobacteria* have an increased abundance, similar to intestinal dysbiosis that characterises IBD, which may be one factor that links these diseases [93,94]. Another factor that links IBD and liver fibrosis is the potential use of *Lactobacillus rhamnosus GG* (*LGG*) as a potential therapeutic agent in both entities of the disease. Liu et al. studied the effect of probiotics as a potential therapeutic agent in liver fibrosis. They showed that *LGG* administration can prevent liver fibrosis by inhibiting liver bile acid synthesis through Farnesoid X receptor; fibroblast growth factor (FXR-FGF-15) mediated signaling in the intestinal tract in animal models [92]. Li et al. showed in vitro that *LGG* can reduce TLR4 expression, indirectly leading to inhibition of nuclear-kappa B factor (NF-κB) activation. The authors indicate that *LGG* should be considered as a potential treatment for IBD [95].

Inflammation in IBD could theoretically lead to the onset and development of liver fibrosis, but the pathophysiology is still unclear and further research is needed in this direction.

## 6. Treatment

The goal of treatment is to achieve and maintain deep remission (healing of the intestinal mucosa), thus reducing the risk of the need for surgical treatment. When considering the implementation of appropriate treatment, the activity and severity of the disease course, the response to previous medications, and concomitant extraintestitial symptoms, among other factors, should be taken into account. Treatment modes can be divided into pharmacological and surgical. An adjunctive component of sublingual treatment is nutritional therapy [96]. Aminosalicylates and glucocorticosteroids (ICS) are the main applications in pharmacological treatment. The group of aminosalicylates includes mesalazine, a 5-ASA monomer, and sulfasalazine. Mesalazine is the treatment of choice during the remission period of UC with the left-sided form, as well as with the presence of rectal inflammation. Systematic intake of 5-ASA during the disease remission period reduces the risk of relapse, as well as colectomy and colorectal cancer [97]. ICS are drugs with anti-inflammatory effects. They stabilise lysosome membranes, reduce prostaglandin synthesis, reduce leukocyte migration, and additionally inhibit T lymphocyte activity. They are the drugs most commonly used (along with 5-ASA preparations) in the induction of clinical remission. ICS is effective in 60–80% of people in the active phase of the disease [98]. Another type of drug is immunosuppressive drugs, including Azathioprine (AZA) and mercaptopurine (MP). Thiopurines induce apoptosis of T lymphocytes by modulating cell signaling. They are used to maintain clinical remission and in cases of resistance to steroids and dependence on steroids [99]. Biological treatment is also used. Drugs obtained by genetic engineering that reduce or disable the activation of certain components of the immune system. They are divided into 3 categories: products similar to naturally occurring substances in the body, monoclonal antibodies, and modified proteins. Treatment with biological agents is used in cases of steroid resistance, intolerance to immunosuppressive drugs, and in the maintenance treatment of clinical remission of the disease [100]. Due to the immunomodulatory functions of mesenchymal stem cells (MSCs), they represent a promising therapeutic option to contribute to the quality of treatment. MSCs, due to their effects, can induce Th17 supression compared to Treg cell responses. They can improve intestinal barrier function and participate in the wound healing process. Several studies show that MSC-based therapy has positive application results in patients with IBD and LF [101,102,103]. Due to the possibility of nutritional deficiencies in patients with IBD and liver disease resulting from malabsorption, chronic inflammation, and/or reduced intake of selected foods, nutritional therapy should be an integral part of treatment. Nutrition therapy is particularly important in patients who are malnourished or have active perioperative inflammation, the main objective of which is to avoid negative nitrogen balance in patients. Support the function of the immune system and shortens recovery time after surgery [104,105].

## 7. Conclusions

Liver disease is very often one of the extraintestinal manifestations of IBD. Due to an inadequate lifestyle, excessive fat and caloric intake, and lack or low physical activity, patients with IBD will often develop additive NAFLD. Through fat accumulation in liver cells and impaired lipid metabolism, NASH can form. Subsequently, HSC activation leads to collagen deposition, predisposing to progression of organ fibrosis. Liver fibrosis in the course of IBD often progresses regardless of the pharmacotherapy provided. Furthermore, patients with Crohn’s disease are at increased risk of developing MAFLD and its consequences, such as LF. Potential factors linking LF and IBD are shared risk factors for MetS and LF, higher prevalence of MAFLD and LF when diagnosed with IBD than in people without IBD, activation of mesenchymal cells in intestinal and liver fibrosis, and bacterial translocation in LF and intestinal fibrosis. However, more research is needed in this direction.

## Figures and Tables

**Table 1:** Intestinal homeostasis disturbances in patients with inflammatory bowel disease [34,35,36,37,38,39,40,41,42,43,44].

Intestinal Epithelial Barrier Disturbances	Intestinal Microbiota Disturbances
-increased expression of claudin-1–reduction of mucus secretion, which increases the risk of enteritis (CD and UC) -increased expression of claudin-2–Increased pores in TJ (CD and UC) -decreased expression of claudin–3,5,8 (CD)–reduction of intestinal barrier properties -decreased expression of claudin–3,4,7 (UC)–reduction of intestinal barrier properties -increased expression of occludin (active UC) -ZO1 dysfunction–decrease in stabilised claudin strands -TNF-α–decrease in normal TJ function and increase in intestinal epithelial apoptosis -IL-1𝛽 i IFN-𝛾–reduction of TJ integrity by affecting occludins and ZO1 -IFN-𝜆–occurrence of Paneth cell defect -dysfunction of ILC3 -secrection of chemokines by EIC–causes and maintains inflammation in IBD (e.g. CXCR1 (+) CXCR2 (+) IL-23)	Increase in the amount of the following bacteria: -*Proteobacteria* (degradation of intestinal mucus) np. *Haemophilus, Pasteurellaceae* *-Streptococcus* *-Fusobacterium* (degradation of intestinal mucus) -*Enterobacteriaceae* np. *E. Coli* (AIEC) Decrease in the amount of the following bacteria: -*Firmicutes* np. *Faecalibacterium prausnitzii, Roseburia* (SCFA producing bacteria) -*Euryarchaeota* -*Bacteroidetes* np. *Prevotella* spp. -*Bifidobacterium* Increase in the amount of following fungus: -*C. albicans* -*C. parapsilosis* -*Aspergillus clavatus* (CD) -*Cryptococcus neoformans* (CD) -*Ascomycota*

CD-Crohn’s Disease, UC-Ulcerative Colitis, TJ-tight junction, ZO-zonula occludens, TNFα-tumor necrosis factor α, IL-1𝛽- Interleukin-1 beta, IFN-𝛾- Interferon gamma, IFN-𝜆-interferon lambda, ILC3-innate lymphoid cells-3, EIC-intestinal epithelial cells, CXCR- CXC chemokine receptor, SCFA-short-chain fatty acids
